# Matrilin-3 Induction of IL-1 receptor antagonist Is required for up-regulating collagen II and aggrecan and down-regulating ADAMTS-5 gene expression

**DOI:** 10.1186/ar4033

**Published:** 2012-09-11

**Authors:** Chathuraka T Jayasuriya, Mary B Goldring, Richard Terek, Qian Chen

**Affiliations:** 1 Department of Orthopedics; Warren Alpert Medical School of Brown University, Providence RI 02903, USA; 2 Research Division, The Hospital for Special Surgery, and Department of Cell and Developmental Biology, Weill Cornell Medical College, New York, NY 10021, USA

**Keywords:** Matrilin-3, interleukin-1, IL-1Ra, collagen 2, aggrecan, MMP-13, ADAMTS-4, ADAMTS-5, chondrocytes, osteoarthritis

## Abstract

**Introduction:**

Deletion or mutation of the gene encoding the cartilage extracellular matrix (ECM) protein matrilin-3 (*MATN3*) results in the early onset of osteoarthritis (OA), suggesting chondroprotective properties of MATN3. To understand the mechanisms underlying these properties, we determined the effects of MATN3 protein on the expression of several key anabolic and catabolic genes involved in chondrocyte homeostasis, and the dependence of such regulation on the anti-inflammatory cytokine: IL-1 receptor antagonist (IL-1Ra).

**Methods:**

The effects of recombinant human (rh) MATN3 protein were examined in C28/I2 immortalized human chondrocytes, primary human chondrocytes (PHCs), and primary mouse chondrocytes (PMCs). Messenger RNA levels of *IL-1Ra*, *COL2A1*, *ACAN*, *MMP-13*, and *ADAMTS-4 *and *-5 *were determined using real-time RT-PCR. Knocking down *IL-1Ra *was achieved by siRNA gene silencing. IL-1Ra protein levels were quantified by ELISA and the Bio-Plex Suspension Array System. COL2A1 protein level was quantified using Western blot analysis. Statistic analysis was done using the two-tailed t-test or one-way ANOVA.

**Results:**

rhMATN3 protein induced gene expression of *IL-1Ra *in C28/I2 cells, PHCs, and PMCs in a dose- and time-dependent manner. Treatment of C28/I2 cells and PHCs with MATN3 protein stimulated gene expression of *COL2A1 *and *ACAN*. Conversely, mRNA levels of *COL2A1 *and *ACAN *were decreased in *MATN3 *KO mice. MATN3 protein treatment inhibited IL-1β-induced *MMP-13*, *ADAMTS-4 *and *ADAMTS-5 *in C28/I2 cells and PHCs. Knocking down *IL-1Ra *abolished the MATN3-mediated stimulation of *COL2A1 *and *ACAN *and inhibition of *ADAMTS-5*, but had no effect on MATN3 inhibition of *MMP-13 *mRNA.

**Conclusion:**

Our findings point to a novel regulatory role of MATN3 in cartilage homeostasis due to its capacity to induce *IL-1Ra*, to upregulate gene expression of the major cartilage matrix components, and to downregulate the expression of OA-associated matrix-degrading proteinases in chondrocytes. The chondroprotective properties of endogenous MATN3 depend partly on its induction of IL-1Ra. Our findings raise a possibility to use rhMATN3 protein for anti-inflammatory and chondroprotective therapy.

## Introduction

Matrilin-3 (*MATN3*) is one of the four members of the matrilin family of noncollagenous oligomeric ECM proteins [1-4]. As the smallest member of this family, it contains a single Von Willebrand Factor A (vWFA) domain, four epidermal growth factor (EGF)-like domains, and an alpha-helical oligomerization domain, which allows it to form oligomers with itself or other matrilin molecules [5]. As an extracellular matrix (ECM) protein, MATN3 was thought to play a major structural role in forming a filamentous matrix network by interacting with collagen fibrils, multiple proteoglycans, and other glycoproteins [5]. Mutations in human *MATN3 *are associated with a variety of skeletal diseases including multiple epiphyseal dysplasia (MED), spondylo-epi-metaphyseal dysplasia (SEMD), and osteoarthritis (OA) [6-9], underscoring its importance in cartilage development and homeostasis. Deletion of the *MATN3 *gene in mice results in no gross skeletal deformity at birth; it does however, cause acceleration of cartilage degeneration during aging [10]. Furthermore, *MATN3 *gene expression is increased in articular cartilage tissues from OA patients [11].

OA is characterized as a disregulation of cartilage homeostasis due to excessive upregulation of catabolic factors and the inability of the chondrocytes to adequately repair the degraded matrix, resulting ultimately in degeneration of the major cartilage ECM components such as type II collagen fibrils and aggrecan [12-15]. ECM catabolism is largely mediated by the matrix metalloproteinase (MMP) family of collagenases, including MMP-13, and by the ADAMTS family of aggrecanases (ADAMTS-4 and -5) [14-21]. They are often expressed in chondrocytes in response to major inflammatory cytokines such as IL-1β produced by the synovium and other joint tissues [12]. Furthermore, there is a correlation between the increased levels of these catabolic enzymes and inflammatory mediators such as prostaglandins, nitric oxide (NO), and pro-inflammatory cytokines such as IL-1β and TNF-α in synovial fluids and joint tissue. Previous studies have implicated IL-1β as one of the major inflammatory cytokines associated with cartilage damage [12,22-25] due to its ability to induce or upregulate the expression of proteinases, including MMPs, plasminogen activator, and aggrecanases [25-30], and to downregulate the expression of endogenous proteinase inhibitors (for example, certain TIMPs) [22,31] and cartilage matrix components such as COL2A1 and ACAN [32-34]. IL-1 stimulates production of pro-inflammatory factors including prostaglandins, leukotrienes and itself [12,35,36]. Importantly, IL-1, IL-1 receptor, and MMPs are expressed by chondrocytes in OA cartilage and can be immunolocalized to the same regions [37-40]. Inhibition of the IL-1β pathway presents a promising means of preventing cartilage degradation during OA pathogenesis. One of the major endogenous inhibitors of the IL-1 pathway is IL-1 receptor antagonist (IL-1Ra) [41-43].

In this study we aimed to test whether MATN3 can positively regulate cartilage homeostasis genes, including those downstream of IL-1, in a manner that can help explain its chondroprotective function. Here we report for the first time several novel regulatory functions of MATN3 including induction of *IL-1Ra*, stimulation of *COL2A1 *and *ACAN *expression, and inhibition of *MMP-13 *and *ADAMTS-4 *and *-5 *expression. We also tested whether these novel regulatory properties of MATN3 depend on its induction of *IL-1Ra*.

## Methods

### MATN3 knockout animals

For the purpose of comparing *Col2a1 *and *Acan *expression between wild-type and *MATN3 *knockout (KO) mice of the same C57BL/6J genetic background, mRNA was extracted from the whole hind limbs of these mice at embryonic day 18.5. Generation of *MATN3 *KO mice have previously been described [10]. An in-frame stop codon was introduced into the second exon of their *MATN3 *gene via homologous recombination during the embryonic stem cell stage, rendering these mice functionally MATN3-null animals. All experiments were performed with the approval of the Rhode Island Hospital Institutional Animal Care and Use Committee (IACUC, number 0231-11).

### Isolation of primary chondrocytes from human and mouse cartilage

Primary mouse chondrocytes (PMCs) were isolated from the rib cages of 6-day-old mice. Primary human chondrocytes (PHCs) were isolated from normal-looking areas of knee articular cartilage obtained from five patients undergoing total joint replacement surgery. Cartilage samples were individually handled and processed separately to isolate chondrocytes. Each cartilage sample was washed twice with sterile PBS within 2 hours of tissue collection and diced into small fragments. Cartilage fragments were digested in 5.0 ml of Pronase (Roche, Indianapolis, IN, USA) in Hank's Balanced Salt Solution (HBSS) at a concentration of 2.0 mg/ml for 30 minutes at 37°C under shaking conditions. The digestion solution was removed and cartilage was washed twice with DMEM medium (Life Technologies, Grand Island, NY, USA). After a second wash, DMEM was removed and replaced with 5.0 ml of Type IA Crude Bacterial Collagenase (Sigma-Aldrich, St. Louis, MO, USA) at 1.0 mg/ml for 8 hours at 37°C under shaking conditions. Collagenase enzyme reaction was stopped by adding 5.0 ml of DMEM medium containing 10% fetal bovine serum (FBS) (Life Technologies) into the digestion mix. Solution was filtered using a 100 μm nylon cell strainer (BD, Franklin Lakes, NJ, USA) to remove clumps, followed by centrifugation at 1500 rpm to pellet the successfully isolated chondrocytes. Pellet was washed twice with DMEM medium and cells were counted using a hemocytometer. Finally, chondrocytes were plated at high density (4.0 × 10^6 ^cells) in 60-mm cell culture dishes using DMEM medium supplemented with 10% FBS and 0.2% Streptomycin (Life Technologies). All studies involving patient tissue samples were conducted in accordance with the Institutional Review Board (IRB) of Rhode Island Hospital. The need to obtain patient consent for the collection of cartilage tissue after surgery was waived by the Rhode Island Hospital IRB.

### Chondrocyte cell culture studies

Cell culture experiments were conducted using PMCs, PHCs, and C28/I2 immortalized human chondrocytes, and involved first plating cells in 6-well culture plates at 200,000 viable cells per well in 1:1 DMEM/F-12 media (Life Technologies) supplemented with 10% FBS. After 48 hours, the culture medium was replaced with 2.0 ml of serum-free 1:1 DMEM/F-12 and incubated for 5 hours prior to treatment with recombinant human (rh) MATN3 protein (100 ng/ml or 200 ng/ml) (R&D Systems, Minneapolis, MN, USA) and/or rhIL-1β (5.0 ng/ml) (PeproTech, Rocky Hill, NJ, USA) for 8 to 36 hours, unless otherwise stated. To optimize the induction of catabolic proteases (that is, MMP-13, ADAMTS family) by IL-1 in both primary and immortalized chondrocytes, we used serum-free media for the duration of the treatment period as in a previous study [44].

### Small interfering RNA-based silencing of IL-1Ra in PHCs

PHCs were transfected for 48 hours at approximately 40 to 50% cell confluency with a small interfering RNA (IL1RN ON-TARGETplus siRNA) (Dharmacon, Chicago, IL, USA) using Lipofectamine 2000 (Invitrogen, Carlsbad, CA, USA) according to the manufacturer's instructions. The siRNA targets and suppresses the mRNA and protein expression of all four endogenous isoforms of human IL-1Ra. A non-silencing siRNA (Allstars Negative Control siRNA) (Qiagen, Valencia, CA, USA) was used as control. PHCs were plated at 200,000 viable cells per well in 6-well culture plates 24 hours prior to siRNA transfection procedure. For each well, 40 pmol of siRNA and 2.0 uL of Lipofectamine 2000 were utilized for transfection. The 40 pmol of siRNA was first diluted in 100 uL of Opti-MEM I Reduced Serum Media. Likewise, 2.0 uL of Lipofectamine 2000 was diluted in 100 uL of Opti-MEM I Reduced Serum Media. After 5 minutes at room temperature, the siRNA and lipid mediator solutions were gently mixed, incubated at room temperature for 20 minutes, and then added to cell culture wells containing 1.0 ml of incomplete DMEM media. Cells were placed in a 37°C incubator and media was changed after 8 hours. PHCs were treated with MATN3 and/or IL-1β 48 hours post transfection for subsequent cell culture experiments.

### Gene expression analysis

Total RNA was isolated from PMCs, PHCs, C28/I2 cells and the hind limbs of embryonic day 18.5 wild-type and *MATN3 *KO mice on the C57BL/6J genetic background [10] using the RNAqueous Kit (Ambion, Austin, TX, USA) according to the manufacturer's instructions. Gene expression analysis was conducted by real time quantitative PCR (RT-qPCR) with the DNA Engine Opticon 2 (Bio-Rad, Hercules, CA, USA) using the QuantiTect SYBR Green PCR kit (Qiagen). For RT-qPCR, 0.5 ug of RNA was reverse-transcribed using iScript cDNA Synthesis Kit (Bio-Rad) according to the manufacturer's instructions. The cDNA of each sample was subjected to RT-qPCR using species-specific primer pairs for genes encoding *IL-1Ra*, *type II collagen*, *aggrecan*, *MMP-13*, and soluble *(s)IL-1Ra*. Exact primer sequences can be found in Table 1. Relative transcript levels were calculated using the delta-delta Ct (ΔΔCt) method, normalized to rRNA 18S expression according to the following equation: X = 2 ^-ΔΔCt^, in which ΔΔCt = (Ct_Exp _- Ct_18S_) - (Ct_Ctl _- Ct_18S_) and X = Relative transcript; Ct_Ctl _= Ct of control group.

**Table 1:** Forward and reverse primer sequences used for real-time PCR

**Species**	**Gene**	**Forward sequence**	***Reverse sequence***
Human	ADAMTS-4	5'-CCCCAGACCCCGAAGAGCCA-3'	*5'-CCCGCTGCCAGGCACAGAAG-3'*
Human	ADAMTS-5	5'-GGCCGTGGTGAAGGTGGTGG-3'	*5'-GCTGCGTGGAGGCCATCGTC-3'*
Human	ACAN	5'-ACCAGACGGGCCTCCCAGAC-3'	*5'-TGGCTCTGCCCCAGAGGGAC-3'*
Human	COL21A	5'-TGAGGGCGCGGTAGAGACCC-3'	*5'-TGCACACAGCTGCCAGCCTC-3'*
Human	IL-1Ra (pair 1)*	5'-CCCGTGAAGGAGAGCCCTTCATTTG-3'	*5'-ACTTTCACCATCATTTCACAAATGCAG-3'*
Human	IL-1Ra (pair 2)**	5'-TGTTCCATTCAGAGACGATCTGCCG-3'	*5'-GAGCATGAGGCTCAATGGGTACC-3'*
Human	MMP-13	5'-ATGCGGGGTTCCTGATGTGG-3'	*5'-GGCCCAGGAGGAAAAGCATG-3'*
			
Murine	Acan	5'-CAGTGCGATGCAGGCTGGCT-3'	*5'-CCTCCGGCACTCGTTGGCTG-3'*
Murine	Col2a1	5'-CACACTGGTAAGTGGGGCAAGACCG-3'	*5'-GGATTGTGTTGTTTCAGGGTTCGGG-3'*
Murine	IL-1Ra	5'-ACCCATGGCTTCAGAGGCAGC-3'	*5'-GCCCCCGTGGATGCCCAAG-3'*

**Figure 1:**
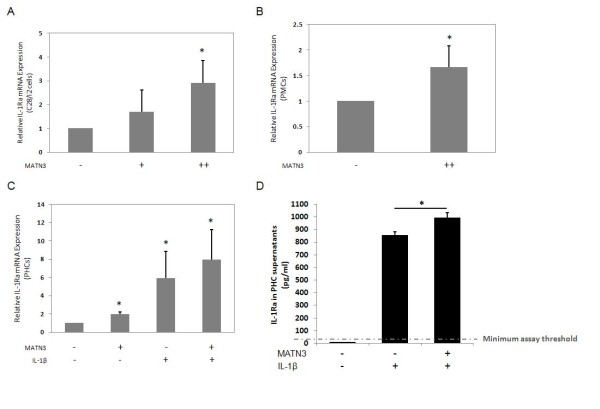
**Matrilin-3 MATN3) enhances IL-1Ra expression by chondrocytes**. Recombinant human (rh) MATN3 protein enhances the gene expression of *IL-1Ra *in a dose-dependent manner at 12 hours post cell culture treatment as seen here in C28/I2 cells (**A**). Recombinant MATN3 also enhances *IL-1Ra *expression in primary mouse chondrocytes after 24 hours of treatment (**B**). MATN3-induced *IL-1Ra *gene expression is evident in primary human chondrocytes (PHCs) at 24 hours post treatment in both the presence and absence of IL-1β (**C**). PHCs treated with both MATN3 and IL-1β for 24 hours exhibit significantly higher concentrations of IL-1Ra protein in their cell media, relative to the media of cells treated with IL-1β alone (**D**). In these experiments, rh MATN3 protein is used at one of two concentrations: lower dose (+) of 100 ng/ml, or higher dose (++) of 200 ng/ml. Rh IL-1β protein treatment is always 5.0 ng/ml. *Significant differences (*P *≤ 0.05) from the untreated control group; ^#^significant differences (*P *≤ 0.05) from the IL-1β only treated group. Data are representative of three individual experiments.

*Primer pair used for IL-1Ra expression analysis in C28/I2 immortalized human chondrocytes and primary human chondrocytes for Figure 1A, 1C and Additional file 1. **Primer pair used for IL-1Ra expression analysis in primary human chondrocytes for Figure 4A and 4B. ACAN, aggrecan; COL2A1, type II collagen; IL-1Ra, interleukin-1 receptor antagonist; MMP-13, matrix metalloproteinase-13.

### Protein analysis

The Human IL-1ra/IL-1F3 Immunoassay (R&D Systems) and the MMP-13 Human ELISA Kit (Abcam, Cambridge, MA) were used to quantify IL-1Ra and MMP-13 protein levels, respectively, in PHCs 24 hours following treatment with MATN3 and/or IL-1β, according to the manufacturer's instructions. PHCs were seeded at a cell density of 100,000 cells/well in 12-well cell culture plates. After 24 hours, media were changed to be serum-free and each group was treated with MATN3 and/or IL-1β as appropriate. Conditioned media were collected 24 hours later, spun down to remove any cell debris and immediately frozen down at -80°C until assays were ready to be performed. The minimum detectable level of IL-1Ra and MMP-13 by these assays were 30 pg/ml and 6.0 pg/ml, respectively.

The Bio-Plex Suspension Array System (Bio-Rad) was used to measure soluble IL-1Ra protein levels in cell culture medium of PHCs transiently transfected with either an siRNA against *IL-1Ra *or a non-silencing scrambled control siRNA. Transiently transfected PHCs were collected 36 hours following treatment with MATN3 and/or IL-1β. These samples were analyzed using Bio-Plex Pro Human Cytokine Assay in duplicate per each biological replicate (two per treatment group) according to the manufacturer's instructions. Protein quantification of type II collagen was conducted via western blot analysis using standard protocols. Prior to Western blot analysis of type II collagen protein, primary human chondrocytes transfected with either the *IL-1Ra*-silencing siRNA or the non-silencing scrambled control siRNA, were seeded and cultured for 48 hours in DMEM containing 10% FBS in the presence and absence of rhMATN3 (200 ng/ml) and/or IL-1β (5.0 ng/ml). Cell culture pellets were resuspended in 50 uL of RIPA buffer (Cell Signaling Technology, Boston, MA, USA) containing protease inhibitors. Protein concentrations were determined using a Bradford Assay (Bio-Rad), according to the manufacturer's instructions, and a NanoDrop 2000c (Thermo Fisher Scientific, Rockford, IL, USA). Western blot analysis was performed using standard protocols. A previously characterized mAb against collagen II [45,46] (NeoMarkers, Fremont, CA, USA) was used as the primary antibody. Beta-actin was used as a loading control for normalization. Imaging was done using the Odyssey Infrared Imager (LI-COR Biosciences, Lincoln, NE, USA). Western band intensities were quantified using ImageJ software program (NIH, Bethesda, MD, USA).

### Statistical analysis

**Figure 2:**
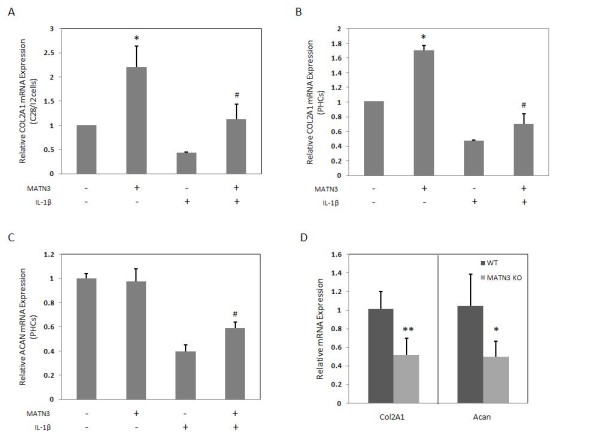
**Matrilin-3 (MATN3) maintains the expression of key cartilage extracellular matrix (ECM) genes**. Recombinant human (rh) MATN3 treatment maintains type II collagen (*COL2A1*) gene expression in the presence and absence of IL-1β in C28/I2 cells (**A**) and primary human chondrocytes (PHCs) (**B**) at 8 hours. MATN3 also inhibits the IL-1β-induced downregulation of aggrecan (*ACAN*) gene expression in PHCs at 36 hours (**C**). Whole limb mRNA analysis of newborn *MATN3 *knockout (KO) mice of the C57BL/6J background reveal lower basal gene expression of *Col21a *and *Acan *compared to wild-type mice of the same genetic background (**D**). For all cell culture experiments, rh MATN3 protein is used at 200 ng/ml. Rh IL-1β protein treatment is always 5.0 ng/ml. *Significant differences (*P *≤ 0.05) from the untreated control group; ^#^significant differences (*P *≤ 0.05) from the IL-1β only treated group. For cell culture experiments, data are representative of three individual experiments (*n *= 5 for mouse whole limb mRNA studies).

Mean values were calculated and are presented with error bars representing ± SDM (one standard deviation of the mean). Two-tailed *t*-tests were used to analyze data represented in Figure 1B and 2D. For all else, statistical analysis was done using one-way analysis of variance (ANOVA) followed by post hoc test analysis. Statistical significance was accepted at *P *< 0.05 for all analyses.

## Results

### MATN3 induces gene expression of IL-1Ra

IL-1Ra is a potent endogenous inhibitor of the IL-1 pathway [37,38]. To determine whether MATN3 affects IL-1Ra synthesis, we treated immortalized human C28/I2 chondrocytes with rhMATN3 protein. MATN3 induced mRNA expression of *IL-1Ra *in a dose-dependent manner (Figure 1A). We also observed an increase of *IL-1Ra *mRNA levels in PMCs treated with rhMATN3 protein (Figure 1B). RhMATN3 protein induced *IL-1Ra *mRNA in PHCs in the presence or absence of IL-1β (Figure 1C). Treating PHCs with MATN3 in the presence of IL-1β significantly enhanced IL-1Ra protein concentration in culture medium compared to that of cells treated with IL-1β alone (Figure 1D). The kinetics of *IL-1Ra *mRNA induction by MATN3 in C28/I2 cells and PHCs was also analyzed (see Additional file 1). The induction of *IL-1Ra *mRNA persisted during a 24-hour treatment period with more pronounced induction in the shorter incubation period. Thus, MATN3 induces the mRNA and protein expression of IL-1Ra in chondrocytes.

### MATN3 stimulates expression of COL2A1 and ACAN

To investigate whether MATN3 stimulates type II collagen gene (*COL2A1*) expression in chondrocytes, we treated C28/I2 cells (Figure 2A) and PHCs (Figure 2B) with rhMATN3 protein for 8 hours. We observed a significant induction of *COL2A1 *expression in these chondrocytes. MATN3 induction of *COL2A1 *was also observed after 24 hours treatment (see Additional file 2). While treatment with IL-1β significantly decreased *COL2A1 *mRNA levels in both C28/I2 cells and PHCs, treatment with MATN3 at the same time reversed this decrease (Figure 2A,B). MATN3 treatment also increased aggrecan gene (*ACAN*) expression by PHCs in the presence of IL-1β (Figure 2C).

To determine whether the lack of *MATN3 *affects the expression of *COL2A1 *and *ACAN *in chondrocytes, we analyzed RNA isolated from the limbs of *MATN3 *KO and wild-type mice. The *MATN3 *KO mice exhibited 50% reduction of *Col2a1 *and *Acan *mRNA levels compared to their wild-type littermates (Figure 2D) indicating that the absence of *MATN3 *suppresses the expression of these two genes.

### MATN3 inhibits gene expression of MMP-13, ADAMTS-4 and ADAMTS-5

**Figure 3:**
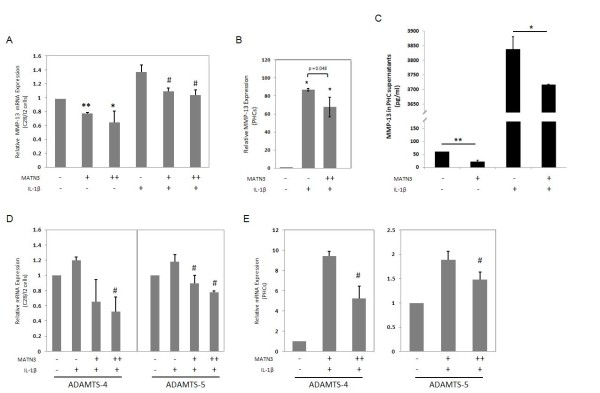
**Matrilin-3 MATN3) inhibits the expression of several osteoarthritis (OA)-associated proteases**. Recombinant human (rh) MATN3 protein inhibits the gene expression of metalloproteinase (MMP)-13 in a dose-dependent manner in the presence and absence of IL-1β in C28/I2 cells (**A**). MATN3 inhibits IL-1β-induced *MMP-13 *mRNA levels (**B**) and protein levels in primary human chondrocyte (PHC)-conditioned media (**C**). MATN3 inhibits the mRNA expression of OA-associated proteases *ADAMTS-4 *and *ADAMTS-5 *in C28/I2 cells (**D**) and PHCs (**E**). *MMP-13 *mRNA and protein analysis were conducted after MATN3 treatment for 24 hours. In these experiments, rh MATN3 protein is used at one of two concentrations: lower dose (+) of 100 ng/ml, or higher dose (++) of 200 ng/ml. Rh IL-1β protein treatment is always 5.0 ng/ml. *P ≤ 0.05, **P ≤ 0.01, for statistically significant differences from the untreated control group; ^#^*P *≤ 0.05, ^##^*P *≤ 0.01 for statistically significant differences from the IL-1β only treated group. Data are representative of three individual experiments.

We next examined whether MATN3 could inhibit the expression of IL-1β-induced protease genes. In C28/I2 cells, IL-1β treatment increased *MMP-13 *mRNA levels and MATN3 treatment decreased *MMP-13 *expression in both the presence and absence of IL-1β (Figure 3A). IL-1β treatment also increased *MMP-13 *gene expression by PHCs and elevated *MMP-13 *mRNA levels and protein concentrations in PHC-conditioned media while treatment with MATN3 abrogated this increase significantly (Figure 3B, C). Likewise, the IL-1β-induced *ADAMTS-4 *and *-5 *gene expression was inhibited by MATN3 in C28/I2 cells (Figure 3D), and in PHCs (Figure 3E).

### IL-1Ra mRNA and soluble protein levels are diminished by IL1Ra siRNA

**Figure 4:**
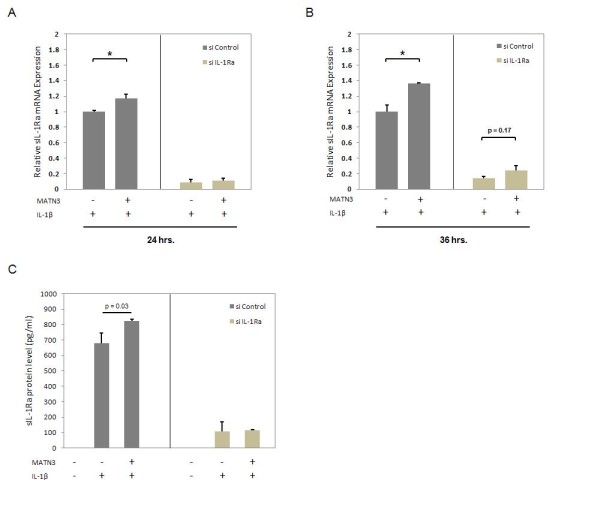
**Small interfering RNA significantly suppresses mRNA and protein levels of IL-1Ra**. Knocking down all isoforms of IL-1Ra via IL1RN siRNA treatment reduces the IL-1β induction of this gene as observed at two time points (24, 36 hours) post cell culture treatment (**A**, **B**). This also results in significant reduction of soluble IL-1Ra protein levels in primary human chondrocyte (PHC) cell supernatants after 24 hours (**C**). PHCs treated with a nonspecific scrambled siRNA construct is used as the control group. For these experiments, recombinant human (rh) matrilin-3 (MATN3) protein is used at 200 ng/ml. Rh IL-1β protein treatment is always 5.0 ng/ml. *P ≤ 0.05 for statistically significant differences between groups. Data are representative of three individual experiments.

Previous studies have shown that IL-1Ra antagonizes IL-1β stimulation of catabolic gene expression and its inhibition of anabolic gene expression. We hypothesize that the chondroprotective properties of MATN3 are dependent on its upregulation of *IL-1Ra*. To test whether MATN3 acts through IL-1Ra to regulate the expression of anabolic and OA-associated catabolic genes, we first knocked down all isoforms of IL-1Ra using a small interfering RNA (siRNA). Since the endogenous soluble IL-1Ra protein levels present in C28/I2 cell supernatants were too low to accurately quantify, all knock-down experiments were conducted in PHCs. The siRNA transfection successfully knocked down both *IL-1Ra *mRNA (Figure 4A, B) and protein expression (Figure 4C) in PHCs, both in the presence and absence of IL-1β.

### MATN3 stimulation of COL2A1 and ACAN depends on IL-1Ra

**Figure 5:**
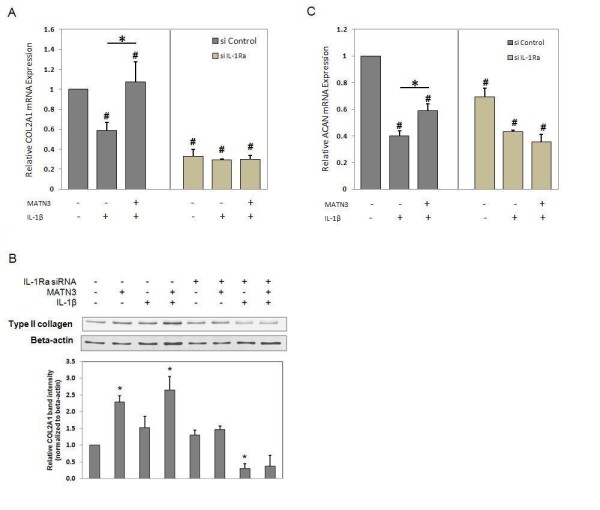
**Matrilin-3 MATN3) regulation of type II collagen (COL2A1) and aggrecan (ACAN) gene expression, as well as COL2A1 protein expression requires IL-1Ra**. Knocking down *IL-1Ra *abolishes the ability of recombinant human (rh) MATN3 protein to maintain *COL2A1 *gene expression in primary human chondrocytes (PHCs) that are challenged with IL-1β (**A**). A significant reduction in the basal gene expression of *COL2A1 *is also observed in *IL-1Ra *knock-down PHCs. PHCs cultured for 48 hours in media supplemented with rh MATN3 protein (200 ng/ml) exhibit increased *COL2A1 *protein levels; however, upon suppressing *IL-1Ra *via siRNA-based gene knock-down, MATN3 enhancement of collagen is diminished according to western blot analysis (**B**). Relative quantification of band intensity is the accumulated result of three individual experiments (*n *= 3). Knocking down of *IL-1Ra *also abolishes MATN3 stimulation of *ACAN *gene expression (**C**). The concentration of rh MATN3 protein used was 200 ng/ml and of IL-1β was 5.0 ng/ml for all experiments. (**A**, **C) **Gene expression analysis was conducted 36 hours post exposure to cell culture treatment conditions. ^#^*P *≤ 0.05 for statistically significant differences from the untreated group; *P ≤ 0.05 for statistically significant differences between groups. (B) *P ≤ 0.05 for statistically significant differences from the untreated control group. Individual experiments were done in biological triplicate per patient sample. Data are representative of five individual experiments.

We then determined the effect of MATN3 on *COL2A1 *expression in *IL-1Ra *knocked-down PHCs. In the control cells, which were transfected with a scrambled siRNA construct, MATN3 treatment rescued the IL-1β induced downregulation of *COL2A1 *mRNA (Figure 5A, left). COL2A1 protein levels were also enhanced by MATN3 treatment in both the absence and presence of IL-1β (Figure 5B). In contrast, MATN3 was incapable of enhancing *COL2A1 *mRNA (Figure 5A, right) or protein levels (Figure 5B) in cells transfected with the IL-1Ra siRNA. Similarly, MATN3 reduced the extent of IL-1β-induced downregulation of *ACAN *mRNA expression in cells transfected with the scrambled siRNA construct (Figure 5C, left) but not in cells transfected with the IL-1Ra siRNA (Figure 5C, right).

### MATN3 inhibition of ADAMTS-5, but not of MMP-13, depends on IL-1Ra

**Figure 6:**
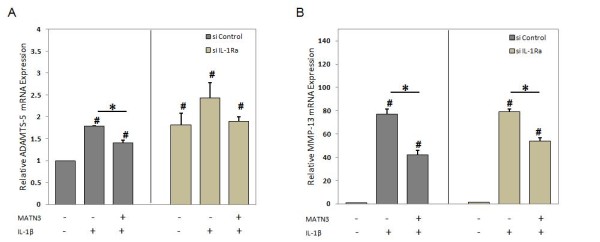
**Matrilin-3 MATN3) inhibition of ADAMTS-5 gene expression require IL-1Ra**. Knocking down *IL-1Ra *in primary human chondrocytes (PHCs) only partially affects MATN3-induced inhibition of *ADAMTS-5 *(**A**) and does not seem to significantly affect *MMP-13 *gene expression (**B**). The concentration of recombinant human MATN3 protein used was 200 ng/ml and of IL-1β was 5.0 ng/ml. Gene expression analysis was conducted 36 hours post exposure to cell culture treatment conditions. ^#^*P *≤ 0.05 for statistically significant differences from the untreated group; *P ≤ 0.05 for statistically significant differences between groups. Individual experiments were done in biological triplicate per patient sample. Data are representative of five individual experiments.

To test whether MATN3 inhibition of matrix proteases is dependent on IL-1Ra, we quantified their expression levels in the *IL-1Ra *knock-down chondrocytes. Knocking down *IL-1Ra *chondrocytes significantly increased the basal level of *ADAMTS-5 *expression (Figure 6A, compare conditions in the absence of MATN3 or IL-1β). The presence of MATN3 significantly inhibited the upregulation of *ADAMTS-5 *by IL-1β (Figure 6A, left panel, *P *≤ 0.05). However, this inhibition was not significant in *IL-1Ra *knock-down chondrocytes (Figure 6A, right panel, *P *= 0.37). Thus MATN3 inhibition of *ADAMTS-5 *is at least partly dependent on IL-1Ra. In contrast, knocking down *IL-1Ra *did not increase the basal level of *MMP-13 *gene expression (Figure 6B). In addition, MATN3 inhibition of *MMP-13 *gene expression was significant in both control and *IL-1Ra *knock-down cells suggesting that MATN3 inhibition of *MMP-13 *is not mediated by IL-1Ra.

## Discussion

Mutations in human *MATN3 *are associated with a variety of human cartilage degenerative diseases including chondrodysplasia and OA [6-9]. These genetic studies strongly suggest that the normal *MATN3 *gene product is chondroprotective. Since MATN3 protein is an ECM protein, an accepted hypothesis is that its chondroprotective activity is due to its structural properties, which may help to maintain tissue integrity [47]. In this study however, we hypothesized that MATN3 may also be capable of preventing the IL-1β-induced disregulation of cartilage homeostasis genes thereby preventing the hallmark of OA development.

Here we demonstrated that rhMATN3 protein induces gene expression of *IL-1Ra *in three chondrocyte culture models: immortalized human C28/I2 chondrocytes, PHCs and PMCs. MATN3 increased *IL-1Ra *mRNA production in the presence and absence of the inflammatory cytokine IL-1β in a dose- and time-dependent manner. Furthermore, MATN3 treatment increased the soluble IL-1Ra protein levels in the presence of IL-1β in chondrocytes, as determined by ELISA and Bio-Plex Array analysis.

IL-1Ra, an IL-1α/β protein mimic, is a major endogenous inhibitor of the IL-1β pathway [12,41-43]. It binds IL-1R1, thereby preventing IL-1 downstream signaling. IL-1Ra is potentially chondroprotective, since it inhibits IL-1β, which is a major stress and inflammation cytokine that is closely associated with OA pathogenesis. The concentration of soluble IL-1Ra decreases with increasing grades of articular chondral damage in human patients [48]. Knocking down *IL-1Ra *results in the early onset of arthritis in multiple mouse genetic backgrounds. *IL-1Ra *KO mice bred in both BALB/cA and MFIx129 backgrounds developed severe inflammatory arthritis [49]. Conversely, *in vivo IL-1Ra *gene transfer reduces surgically induced OA severity in rabbits [50]. The level of IL-1Ra present in the rabbit synovium positively correlated with the reduction in articular cartilage lesions.

Because of its chondroprotective properties, it is of paramount importance to study how *IL-1Ra *gene expression is regulated. IL-1Ra is produced by many cell types including chondrocytes [51]. It is important to recognize that the chondroprotective effects of IL-1Ra in OA are only observable when the protein is consistently present in the arthritic joint. This explains why short-lived drugs based on IL-1Ra (that is, AnikinRA) [52], which only last a few hours post-intra-articular injection into human patients, have limited efficacy in OA treatment [53]. Thus, finding a means of continuously stimulating autologous IL-1Ra production in chondrocytes may provide an effective alternative for sustaining articular cartilage integrity by dampening inflammation under arthritic conditions. We show here for the first time that MATN3 protein is capable of stimulating *IL-1Ra *expression in chondrocytes. Our findings may also explain a previous report of increased *MATN3 *expression in OA patients [11], since articular chondrocytes may be attempting to inhibit IL-1-induced joint tissue destruction by increasing MATN3 production as a means of recovery.

To further test the hypothesis that MATN3 is chondroprotective, we examined the effect of MATN3 on the expression of anabolic genes such as *COL2A1 *and *ACAN *and catabolic genes such as *ADAMTS-4 *and *-5 *and *MMP-13 *in chondrocytes. Recombinant human MATN3 protein enhanced *COL2A1 *expression while inhibiting the IL-1β-induced downregulation of *COL2A1 *and *ACAN*. Conversely, the lack of MATN3 resulted in reduced basal expression of these chondrogenic genes in *MATN3 *KO mice in comparison to wild-type mice of the same genetic background. The levels of both *Col2a1 *and *Acan *were reduced by approximately 50% compared to wild-type mice. Thus, MATN3 is necessary to maintain normal expression of these genes in cartilage. It is interesting that *MATN3 *KO mice have relatively normal skeletal development [10] despite reduced levels of *Col2a1 *and *Acan *as observed here for the first time. This is consistent with the finding that *Col2a1*and *Acan*heterozygous KO mice have normal skeletal development at birth [54,55]. However, our data also show that, while IL-1β treatment greatly reduces the levels of *COL2A1 *and *ACAN *in chondrocytes, the presence of MATN3 inhibits this further reduction of matrix synthesis by IL-1β. Since the IL-1β levels are often elevated in OA, the lack of MATN3 may result in a more severe phenotype during skeletal aging than during development. Thus, this newly discovered MATN3 property of inhibiting IL-1β may explain why the lack of MATN3 results in accelerated OA during aging despite relatively normal skeletal development in *MATN3 *KO mice [10].

MATN3 protein not only maintained expression of anabolic genes including *COL2A1 *and *ACAN *in our experiments, but also inhibited expression of catabolic genes induced by IL-1β, including *MMP-13*, *ADAMTS-4 *and *-5*. This inhibitory effect correlated with the concentration of MATN3 protein in the culture medium. These catabolic genes are closely related to OA pathogenesis with increased gene expression and/or activity [12]. The lack of the *ADAMTS-5 *gene also protects cartilage degeneration from abnormal mechanical loading in the mouse joint [20]. Thus, inhibiting gene expression of these OA-associated matrix proteases induced by IL-1β may also contribute to the chondroprotective properties of MATN3.

Since MATN3 induces *IL-1Ra *expression and inhibits the effects of IL-1β in chondrocytes, we determined whether these two events are dependent. Knocking down *IL-1Ra *using siRNA abolished both *IL-1Ra *mRNA and protein levels in chondrocytes. This was also confirmed by the failure of IL-1β treatment to induce *IL-1Ra *expression in the *IL-1Ra *knocked-down chondrocytes. The levels of *COL2A1 *and *ACAN *mRNA levels were significantly lower in these *IL-1Ra *knocked-down chondrocytes compared to wild-type chondrocytes. Furthermore, treatment with MATN3 protein failed to significantly enhance *COL2A1 *and *ACAN *mRNA levels in the presence of IL-1β. Similarly, western blot analysis indicated that silencing *IL-1Ra *resulted in an overall reduction of COL2A1 protein level. Silencing *IL-1Ra *also completely abolished MATN3 induction of COL2A1 protein level, both in the absence and presence of IL-1β. This suggests that the ability of MATN3 to maintain anabolic markers such as *COL2A1 *and *ACAN *in chondrocytes depends on the presence of IL-1Ra.

Our study reveals that MATN3 inhibition of IL-1β-stimulated expression of catabolic proteases is mediated by both IL-1Ra-dependent and -independent pathways. In the *IL-1Ra *knocked-down cells, *ADAMTS-5 *mRNA levels were significantly increased in comparison to wild-type chondrocytes, while *MMP-13 *mRNA levels remained unchanged. Upon IL-1β treatment, MATN3 inhibition of the stimulation of *ADAMTS-5 *in *IL-1Ra *knocked-down chondrocytes became statistically insignificant. In contrast, MATN3 still significantly inhibited the stimulation of *MMP-13 *by IL-1β in the knocked-down cells as in wild-type chondrocytes. The same was true for *ADAMTS-4 *gene expression as well (not shown). This indicates that MATN3 inhibition of IL-1β upregulation of *ADAMTS-5 *is IL-1Ra-dependent, while its inhibition of IL-1β upregulation of *MMP-13 *and *ADAMTS-4 *is not. In addition to IL-1Ra, there are other antagonists in the IL-1β signaling pathway including a decoy receptor IL-1RII, an inhibitory receptor SIGIRR, and a soluble receptor sIL-1RAcP [56,57]. MATN3 may inhibit IL-1β-stimulation of MMP-13 by upregulating one of these antagonists. Alternatively, MATN3 may also exert its regulatory function by downregulating agonists in the IL-1 signaling pathway that are independent of IL-1Ra. A limitation of this study involves the use of a relatively small patient pool (total of three to five patients) for the acquisition of primary human chondrocytes. The conclusion of MATN3 induction of IL-1Ra is, however, valid in chondrocytes from each patient. Thus, experiments involving the use of primary human chondrocyte cultures accomplished its intended purpose of corroborating the findings observed in the C28/I2 human chondrocyte cell line.

The mechanism by which MATN3 regulates IL-1Ra is currently unknown. Given that *IL-1Ra *mRNA transcript levels are elevated relatively quickly (within 24 hours of exposure to MATN3 recombinant protein), the stimulation by MATN3 of this anti-inflammatory cytokine may be a direct effect. Cartilage extracellular matrix molecules such as hyaluronan have previously been shown to mediate regulatory functions by direct interaction with cell surface molecules and/or receptors [58,59]. It is possible that chondrocytes have an endogenous receptor through which MATN3 signals to mediate its regulatory function(s). It has been shown previously that matrilins may interact with integrins or other plasma membrane receptors in chondrocytes [60]. It remains to be determined whether the regulatory functions of MATN3, as shown here, are mediated by these receptors.

We have shown that MATN3 treatment inhibits OA-associated catabolic gene expression. Interestingly, a previous study has shown that treatment of human chondrocytes with very high concentrations of murine recombinant MATN3 protein leads to activation of OA-associated catabolic genes [61]. It is important to note that there are two clear differences between the aforementioned study and this one. The first is that, while mouse recombinant MATN3 was used to treat PHCs in the previous study, we used human recombinant MATN3 to treat human cells in this study. The second, and perhaps a major difference, is the concentration of recombinant MATN3 used to treat chondrocytes. Recombinant MATN3 protein was used at 5 to 50 μg/ml concentrations in the previous study, which is 50 to 500 times higher than the concentrations (100 to 200 ng/ml) used in this study. Proteins at concentrations significantly higher than the physiological levels may exert toxic effects on cells thereby triggering production of degenerative proteases. Indeed, chondrocytes treated with type II collagen at the same high concentrations also induced matrix protease expression in the previous study [61]. Thus, the data from the two studies indicate that MATN3 is chondroprotective at the 100 to 200 ng/ml concentration range, while stimulating catabolic pathways at the 5 to 50 μg/ml concentration range, which may reflect supra-physiological levels.

## Conclusion

This study presents evidence of several novel regulatory functions of MATN3, including induction of IL-1Ra expression, maintenance of collagen II and aggrecan gene expression, and inhibition of the IL-1β-induced gene expression of certain catabolic matrix proteases including *ADAMTS-5*. These regulatory functions are MATN3 concentration-dependent. We further demonstrate that some of these regulatory functions (that is, enhancement of *COL2A1 *and *ACAN *gene expression) are dependent on IL-1Ra, while other regulatory functions are independent (that is, inhibition of IL-1β-induced *MMP-13 *gene expression). These observations were made in multiple chondrocyte culture models, including primary and immortalized cells, and human and mouse species. It provides a novel mechanism for chondroprotective properties of MATN3, which has been strongly indicated by previous genetic studies in humans and mice. It suggests that MATN3 plays not only a structural role in cartilage ECM, but also a regulatory role in cartilage homeostasis by modulating genes critical for matrix synthesis, degradation, and inflammation. It also raises an intriguing possibility of using MATN3, a cartilage matrix protein, for stimulating endogenous anti-inflammatory and chondroprotective properties in cartilage.

## Abbreviations

ACAN: aggrecan; ANOVA: one-way analysis of variance; COL2A1: type II collagen, DMEM: Dubecco's modified Eagle's medium; ECM: extracellular matrix, EGF: epidermal growth factor; ELISA: enzyme-linked immunosorbent assay; FBS: fetal bovine serum; HBSS: Hank's Balanced Salt Solution; IL-1β: interleukin-1 beta; IL-1Ra: interleukin-1 receptor antagonist; KO: knockout; MATN3: matrilin-3; MED: multiple epiphyseal dysplasia; MMP: matrix metalloproteinase; NO: nitric oxide; OA: osteoarthritis; PBS: phosphate-buffered saline; PHC: primary human chondrocyte; PMC: primary mouse chondrocyte; RT-qPCR: real time quantitative polymerase chain reaction; rh: recombinant human; SEMD: spondylo-epi-metaphyseal dysplasia; sIL-1Ra: soluble interleukin-1 receptor antagonist; siRNA: small interfering ribonucleic acid; vWFA: Von Willebrand Factor A.

## Competing interests

The authors declare that they have no competing interests.

## Authors' contributions

In addition to conducting all cell culture experiments and mRNA/protein analyses, CTJ played a significant role in study design, interpretation and composition of this manuscript. MBG provided the C28/I2 human chondrocyte cell line, which was crucial for this study. She also contributed greatly to data discussion and interpretation. RT is the orthopedic surgeon who extracted the patient cartilage samples that were used for isolating the primary human chondrocytes in this study. He also contributed greatly to data discussion and interpretation. QC (corresponding author) designed all experiments and played a pivotal role in data discussion/interpretation and manuscript composition. This manuscript was read and approved by all aforementioned authors.

## Author's information

CT Jayasuriya, Ph.D candidate of Pathobiology Graduate Program (Brown University); MB Goldring, Ph.D, Professor of Cell and Developmental Biology (Weill Cornell Medical College); R Terek, MD, MA, Associate Professor of Orthopaedics (Warren Alpert Medical School of Brown University); Q Chen, Ph.D, Professor of Medical Science (Brown University).

## Supplementary Material

Additional file 1 **Kinetics of matrilin-3 (MATN3)-induced *IL-1Ra *gene expression in human chondrocytes**. A figure showing MATN3 stimulation of *IL-1Ra *gene upregulation by C28/I2 cells and primary human chondrocytes (PHCs) in the absence (**A**, **C**) and presence of IL-1β (**B**, **D**). Cells were treated with 0, 100 or 200 ng/ml of recombinant human MATN3 protein. IL-1β was used at a concentration of 5.0 ng/ml. *P ≤ 0.05 for statistically significant differences relative to the 0 ng/ml treatment group, for each respective time point. Individual experiments were done in biological triplicate. Click here for file

Additional file 2 **Matrilin-3 (MATN3) stimulates type II collagen (*COL2A1*) mRNA levels for at least 24 hours in human chondrocytes**. A figure showing that MATN3 induces *COL2A1 *mRNA levels in C28/I2 cells (**A**) and primary human chondrocytes (PHCs) (**B**) after 24 hours treatment. Recombinant human (rh) MATN3 protein is used at 200 ng/ml and rh IL-1β protein treatment is used at 5.0 ng/ml. *P ≤ 0.05 for statistically significant differences from the untreated control group; ^#^*P *≤ 0.05 for statistically significant differences from the IL-1β only treated group. Data are representative of three individual experiments. Click here for file
