# Supplementary figures and images for: Matrilin-3 Induction of IL-1 receptor antagonist Is required for up-regulating collagen II and aggrecan and down-regulating ADAMTS-5 gene expression
Source: Arthritis Res Ther. 2012 Sep 11;14(5):R197. doi: 10.1186/ar4033 (PMC3580507; doi:10.1186/ar4033)

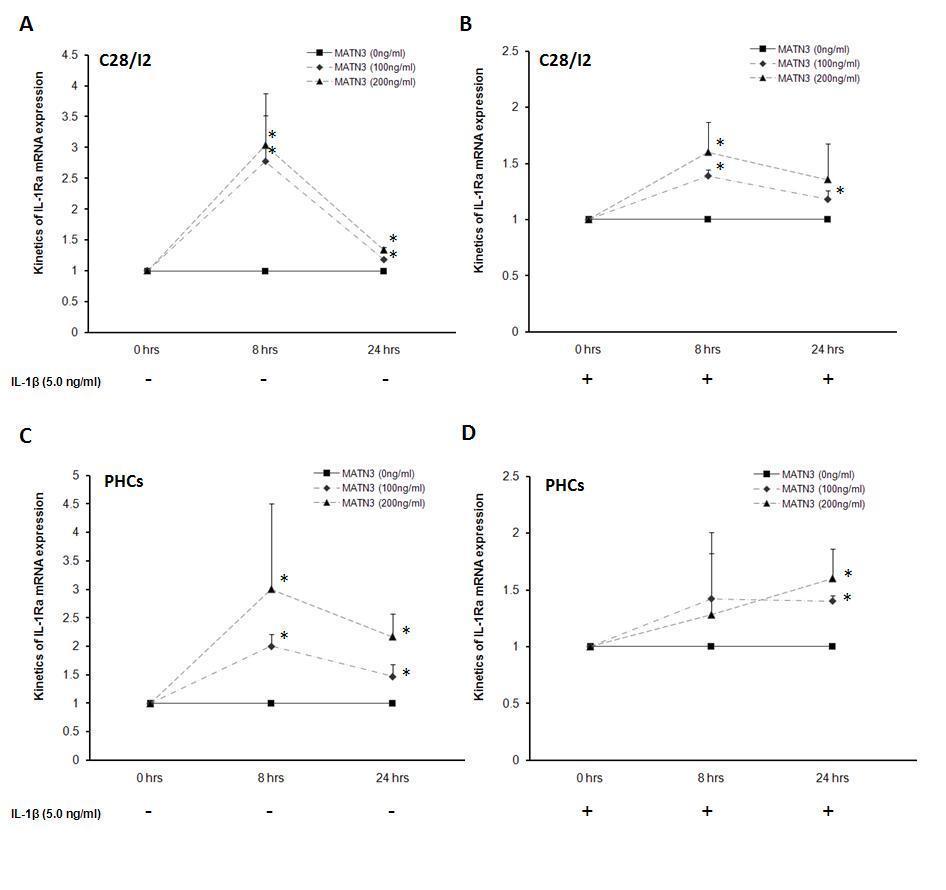

Supplement: Additional file 1 — Kinetics of matrilin-3 (MATN3)-induced IL-1Ra gene expression in human chondrocytes. A figure showing MATN3 stimulation of IL-1Ra gene upregulation by C28/I2 cells and primary human chondrocytes (PHCs) in the absence (A, C) and presence of IL-1β (B, D). Cells were treated with 0, 100 or 200 ng/ml of recombinant human MATN3 protein. IL-1β was used at a concentration of 5.0 ng/ml. *P ≤ 0.05 for statistically significant differences relative to the 0 ng/ml treatment group, for each respective time point. Individual experiments were done in biological triplicate. [file ar4033-S1.JPEG]

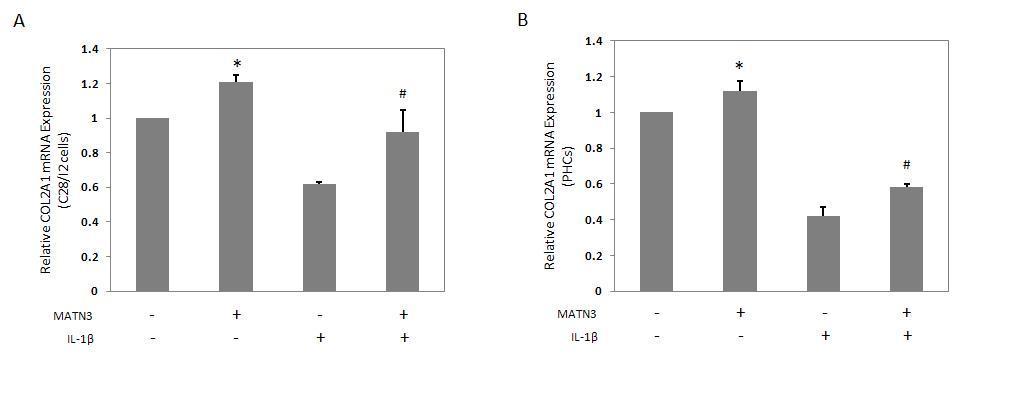

Supplement: Additional file 2 — Matrilin-3 (MATN3) stimulates type II collagen (COL2A1) mRNA levels for at least 24 hours in human chondrocytes. A figure showing that MATN3 induces COL2A1 mRNA levels in C28/I2 cells (A) and primary human chondrocytes (PHCs) (B) after 24 hours treatment. Recombinant human (rh) MATN3 protein is used at 200 ng/ml and rh IL-1β protein treatment is used at 5.0 ng/ml. *P ≤ 0.05 for statistically significant differences from the untreated control group; #P ≤ 0.05 for statistically significant differences from the IL-1β only treated group. Data are representative of three individual experiments. [file ar4033-S2.JPEG]
